# Frailty related all-cause mortality or hospital readmission among adults aged 65 and older with stage-B heart failure inpatients

**DOI:** 10.1186/s12877-021-02072-6

**Published:** 2021-02-16

**Authors:** Pei-Pei Zheng, Si-Min Yao, Wei He, Yu-Hao Wan, Hua Wang, Jie-Fu Yang

**Affiliations:** Department of Cardiology, Beijing Hospital, National Center of Gerontology, Institute of Geriatric Medicine, Chinese Academy of Medical Sciences, No. 1, DaHua Road, Dong Dan, Beijing, 100730 P. R. China

**Keywords:** Frailty, Stage B heart failure, All-cause mortality or readmission, Clinical HF

## Abstract

**Background:**

Frailty increases the adverse outcomes of clinical heart failure; however, the relationship between frailty and stage-B heart failure (SBHF) remains unknown. We aimed to explore the epidemiology and predictive value of frailty in older adults with SBHF.

**Methods:**

A prospective cohort of SBHF inpatients aged 65 years or older who were hospitalized between September 2018 and February 2019 and were followed up for 6 months were included. SBHF was defined as systolic abnormality, structural abnormality (left ventricular enlargement, left ventricular hypertrophy, wall motion abnormalities, valvular heart disease), or prior myocardial infarction. Frailty was assessed by the Fried frailty phenotype. Multivariable Cox proportional hazards regression was used to explore the independent risk and prognostic factors.

**Results:**

Data of 443 participants (age: 76.1 ± 6.79 years, LVEF: 62.8 ± 4.92%, men: 225 [50.8%], frailty: 109 [24.6%]) were analyzed. During the 6-month follow-up, 83 (18.7%) older SBHF inpatients experienced all-cause mortality or readmission, and 29 (6.5%) of them developed clinical HF. Frail individuals had a 1.78–fold (95%CI: 1.02–3.10, *P* = 0.041) higher risk of 6-month mortality or readmission and a 2.83–fold (95%CI 1.24–6.47, *P* = 0.014) higher risk of developing clinical HF, independent of age, sex, left ventricular ejection fraction, and N-terminal pro-B-type natriuretic peptide level.

**Conclusions:**

Frailty is common in older SBHF inpatients and should be considered to help identify individuals with an increased risk of mortality or readmission, and developing clinical HF.

**Trial registration:**

ChiCTR1800017204.

**Supplementary Information:**

The online version contains supplementary material available at 10.1186/s12877-021-02072-6.

## Background

Heart failure (HF) is a highly prevalent, progressive condition and associated with substantial morbidity and mortality. It is an important cause of hospitalization in older adults [1]. Among people > 65 years presenting to primary care facilities with breathlessness on exertion, one in six have unrecognized HF [2]. The prognosis of HF is poor and health care costs are high [1]. Frailty is also highly prevalent in older adults and is associated with a high risk of falling, disability, hospitalization, and mortality [3]. Frailty is a biological syndrome of decreased reserve and resistance to stressors, with the features of weakness, decreased endurance, and slowed performance [4, 5].

Frailty is of particularly concern for patients with HF. HF put the repeatedly exposes geriatric patients to stress and vulnerability, and the prevalence of frailty is higher in these patients than in the general older adults, which may be associated with depression, disability, and cognitive impairment [6–8]. According to the process of HF development [9], HF is divided into four stages of A (preHF), B (preclinical HF), C (clinical HF), and D (refractory HF). Onset of HF may be delayed or prevented by interventions aimed at treating asymptomatic left ventricular dysfunction [10]. Previous studies have focused on frailty and stage C or D HF, but no previous study has explored frailty and stage-B HF (SBHF), which is a clinically reversible stage.

If frailty can also predict poor outcomes, including all-cause mortality or readmission and the onset of clinical HF in older SBHF inpatients, then interventions targeting frailty will provide a new way to improve outcomes in SBHF inpatients. Therefore, we explored the prognostic value of frailty in older SBHF inpatients using the criteria of the Fried frailty phenotype.

## Methods

### Study design

We conducted a prospective study with the cohort derived from our previous study on frailty among older adults [11]. We explored the characteristics and prognostic value (mortality or readmission, and clinical HF) of frailty in older SBHF inpatients.

### Participants

**Fig. 1:**
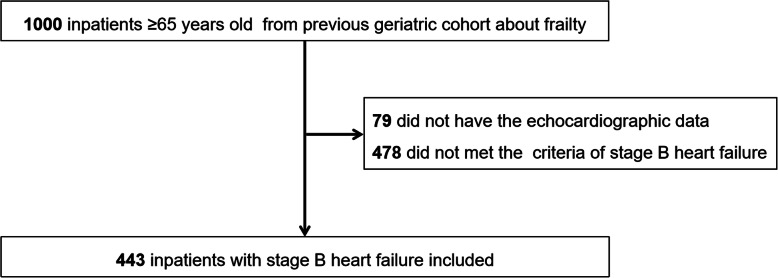
Flow chart of the stage B heart failure cohort

Our previous study on frailty included a geriatric cohort and we surveyed 1000 geriatric patients hospitalized on admission from 10 wards covering the medical and surgical departments of a tertiary referral hospital in Beijing, China, during the period of September 2018 to February 2019 [11]. The inclusion criteria were as follows: hospitalized patients aged 65 years or more, the provision of written informed consent, and the ability to cooperate with the assessment required. Among them, 557 patients were not included in our present cohort because they either did not meet the criteria of SBHF or lacked echocardiographic data. Finally, 443 consecutive, eligible participants were included in this study (Fig. 1).

All participants’ demographic data, SBHF data, diastolic abnormality data, and other characteristics were collected. Participants underwent assessments related to frailty, emotional state, physical function, and cognition immediately after recruitment.

Peking University Clinical Research Institute supervised the study, and the Research Electronic Data Capture (REDCap) [12] was used to manage the acquired data. We collected information through specific investigators, who have been trained in administering our questionnaires.

### Defining SBHF

SBHF refers to patients with structural heart disease that is strongly associated with the development of HF, but without HF signs or symptoms, which includes systolic abnormality, structural abnormality (left ventricular enlargement, left ventricular hypertrophy, wall motion abnormalities, valvular heart disease), and prior myocardial infarction, according to the 2013 ACC/AHA Guidelines for the Evaluation and Management of Chronic HF in the Adult [9], Moreover, we furtherly analyzed the prevalence of diastolic abnormality among the participants. The above echocardiographic data were defined by the criteria of the Atherosclerosis Risk in Communities (ARIC) study, which defined 95% percentile limits derived from a healthy subgroup aged 67–91 years (Table 1).

**Table 1:** The ARIC criteria for wall motion abnormalities, valvular heart disease, LV enlargement, LV hypertrophy, systolic abnormality and diastolic abnormality

	Men	Women
Wall motion abnormalities	Hypokinesis, akinesis, or dyskinesis of two or more contiguous segments of the LV	
Valvular heart disease	Moderate or greater stenosis or regurgitation in the aortic or mitral valve	
LV enlargement	LVEDV/BSA > 60.2 mL/m^2^	LVEDV/BSA > 51.9 mL/m^2^
LV hypertrophy	LV mass/height > 45 g/m^2.7^	LV mass/height > 41.5 g/m^2.7^
Systolic abnormality	LVEF < 59%	LVEF < 57.4%
Diastolic abnormality	LAV/BSA > 34.2 ml/m^2^; septal e’ < 4.3 cm/s; septal E/e’ > 14.8	LAV/BSA > 32.4 ml/m^2^; septal e’ < 4.1 cm/s; septal E/e’ > 17.4

*Abbreviations*: *ARIC* the Atherosclerosis Risk in Communities, *LV* Left ventricular, *LVEDV* Left ventricular end-diastolic volume, *BSA* Body surface area, *LVEF* Left ventricular ejection fraction, *LAV* Left atrial volume

### Frailty assessment

Frailty was evaluated according to the Fried frailty phenotype, defined in the Cardiovascular Health Study according to Fried and colleagues [5]. Five criteria were considered: unintentional weight loss, sense of low energy or exhaustion, muscle weakness, slowed gait, and low physical activity (see Additional file 1). A score of 3 or more was considered to indicate frailty.

### Other assessments

Emotional state was evaluated by the means of the 5-item version of the Geriatric Depression Scale (GDS-5) [13] and anxiety scale was assessed based on the Hospital Anxiety and Depression Scale (HADS-A) [14]. Both of these are validated and extensively used self-reported questionnaires designed for use in the outpatient setting. A GDS-5 score ≥ 2 was considered depression. A HADS-A score ≥ 8 was considered to indicate anxiety.

Activities of daily living were evaluated by the means of the Barthel Index [15], with lower scores indicating increased disability. The maximum score was 100, and the index was used to predict subjects’ dependency needs. A score of < 60 was considered to indicate moderate or severe disability.

Cognition was evaluated by using the Mini Mental State Examination (MMSE) [16]. It is effective for screening for cognitive impairment in older individuals. A score < 24 was considered to indicate cognitive impairment.

### Endpoints

The primary endpoint was 6-month all-cause mortality or hospital readmission. The secondary endpoint was the occurrence of clinical HF. The endpoints were verified by telephone at 6 months after enrollment.

### Statistical analysis

Shapiro–Wilk tests and quantile-quantile plots were used to investigate the normality of data distribution of continuous variables. Frequency distribution, mean ± standard deviation, and median (interquartile range: 25th to 75th percentiles) were used for descriptive analysis of baseline characteristics. The *χ*^*2*^ test, *t-*test, and Mann–Whitney *U* test were utilized to compare the baseline variables by groups (non-frail vs. frail).

Univariate and multivariate logistic regression models were implemented to determine independent risk factors of frailty. All covariates with a *P* value < 0.05 in univariable analysis entered into the multivariable model. The Box–Tidwell test was used to prove the linearity between logitP and continuous independent variables. Tolerance and the variance inflation factor were used to establish that multi–collinearity was not present. Logistic regression analysis results were expressed by odds ratios (ORs) and 95% confidence intervals (95%CIs).

Kaplan–Meier analysis (log-rank test) and multivariate Cox proportional hazards regression (adjusted by age, defined as a categorical variable with a cut off of 80 years; sex; N-terminal pro-B-type natriuretic peptide [NT-pro BNP] level and left ventricular ejection fraction [LVEF]) was utilized to explore the association between frailty and study endpoints in all participants. Similar analyses were performed for the five frailty components about mortality or readmission. The adjusted confounders were selected according to the univariate Cox proportional hazards regression (demographics, characteristics diagnosed as SBHF, diastolic abnormality, LVEF, NT-pro BNP, hypertension, blood pressure controlled, persistent atrial fibrillation/flutter, diabetes mellitus, ≥ 5 medications, beta-blockers, renin-angiotensin system inhibitor, GDS-5 ≥ 2, HADS ≥8, Barthel index < 60, and MMSE < 24, confounders with *P* < 0.1 were selected) and clinical relevance. The results of Cox proportional hazards regression models were expressed by hazard ratios (HRs) and 95%CIs.

SPSS software version 25 (IBM Corp., Armonk, NY, USA) was used for statistical analysis. All statistical tests were two-tailed and a *p*-value < 0.05 was considered to be statistically significant.

## Results

### Baseline characteristics and risk factors of frailty

The demographic and clinical characteristics of the patients categorized as frail and non-frail are shown in Table 2. Frail SBHF inpatients were older, had more comorbidities, a higher level of NT-pro BNP, lower BMI, and lower LVEF. They also had worse blood pressure control and a larger proportion of diastolic abnormalities, polypharmacy, depression, disability, a higher number of falls in the previous year, cognitive impairment and a smaller proportion exercised ≥5 times per week or could walk 100 m without help. Importantly, the prevalence of every component of frailty was extremely higher among the frail participants.

**Table 2:** Demographic and clinical characteristics of all participants

	Overall *n* = 443	Non-frail *n* = 334 (75.4%)	Frail *n* = 109 (24.6%)	*P* values
**Demographics**				
Age, years	76.1 ± 6.79	75.1 ± 6.62	79.1 ± 6.39	**< 0.001**
Man	225 (50.8)	165 (49.4)	60 (55.0)	0.306
Medical insurance	372 (84)	277 (82.9)	95 (87.2)	0.297
Manual workers	156 (30.5)	120 (36.1)	36 (33.0)	0.555
Living with spouse	313 (70.7)	243 (72.8)	70 (62.4)	0.089
Education, years	10.8 ± 4.56	10.9 ± 4.50	10.5 ± 4.72	0.406
Current smoking	41 (9.3)	29 (8.7)	12 (11.0)	0.701
Current drinking	104 (23.5)	86 (27.5)	18 (16.5)	0.137
BMI, kg/m^2^	25.1 ± 3.48	25.4 ± 3.30	24.1 ± 3.83	**0.001**
**Characteristics diagnosed as SBHF**				
Myocardial infarction	62 (14.0)	47 (14.1)	15 (13.8)	0.935
Systolic abnormality	47 (10.8)	32 (9.7)	15 (13.9)	0.226
Structural abnormality	422 (97.0)	318 (97.2)	104 (96.3)	0.229
**Diastolic abnormality**	193 (43.6)	136 (40.7)	57 (52.3)	**0.034**
**LVEF, %**	62.8 ± 4.92	63.1 ± 4.75	62 ± 5.33	**0.046**
**Preserved LVEF, ≥50%**	425 (97.3)	321 (97.6)	104 (96.3)	0.483
**NT-pro BNP, pg/ml**	166 [83.4, 416]	150 [77.7, 344]	322 [123, 655]	**< 0.001**
**Other Characteristics**				
Hypertension	361 (81.5)	271 (81.1)	90 (82.6)	0.738
Blood pressure ≤ 140/90 mmHg	178 (49.3)	141 (52.0)	37 (41.1)	**0.047**
Persistent atrial fibrillation/flutter	30 (41.7)	19 (42.2)	11 (40.7)	0.550
Diabetes mellitus	136 (30.7)	102 (30.5)	34 (31.2)	0.898
≥ 5 medications	220 (49.7)	152 (45.5)	68 (62.4)	**0.002**
Number of comorbidities	3.22 ± 1.46	3.13 ± 1.45	3.48 ± 1.48	**0.032**
Beta-blockers	174 (43.4)	129 (42.3)	45 (46.9)	0.479
RASI	139 (34.7)	110 (36.1)	29 (30.2)	0.326
**Fried frailty components**				
Unintentional weight loss	50 (11.3)	17 (5.1)	33 (30.3)	**< 0.001**
Low physical activity	112 (25.3)	42 (12.6)	70 (64.2)	**< 0.001**
Physical exhaustion	218 (49.2)	125 (37.4)	93 (85.3)	**< 0.001**
Muscle weakness	157 (35.7)	68 (20.5)	89 (82.4)	**< 0.001**
Slow gait	207 (52.7)	128 (41.8)	79 (90.8)	**< 0.001**
**Emotion**				
GDS-5 ≥ 2	51 (11.5)	29 (8.7)	22 (20.2)	**0.001**
HADS ≥8	19 (4.3)	11 (3.3)	8 (7.3)	0.070
**Physical Function**				
Barthel index < 60	21 (4.7)	9 (2.7)	12 (11)	**< 0.001**
Exercise ≥5 per week	331 (74.7)	292 (87.4)	39 (35.8)	**< 0.001**
Walk 100 m without help	361 (81.5)	306 (91.6)	55 (50.5)	**< 0.001**
Fall in the past year	101 (22.8)	65 (19.5)	36 (33.0)	**0.003**
**Cognition,** MMSE < 24	71 (16.0)	35 (10.5)	36 (33.0)	**< 0.001**

Notes: Values are showed as mean ± standard deviation or n (%). Data was analyzed through *t* test for normally distributed continuous data and *χ*^*2*^ test for categorical data

Abbreviations: *BMI* Body mass index, *LVEF* Left ventricular eject fraction, *NT-pro BNP* N-terminal pro-B-type natriuretic peptide, *RASI* Renin-angiotensin system inhibitor, *GDS-5* Five-item version of the Geriatric Depression Scale, *HADS-A* Anxiety scale from Hospital Anxiety and Depression Scale, *MMSE* Mini Mental State Examination

Polypharmacy (ORs 2.24, 95%CIs 1.21–4.15, *P* = 0.010), disability (ORs 4.88, 95%CIs 1.05–22.63, *P* = 0.043) and cognitive impairment (ORs 4.14, 95%CIs 2.09–8.22, *P* = 0.035) were independently associated with frailty in SBHF as shown in more detail in the additional file (see Additional file 2).

### Mortality or readmission

**Fig. 2:**
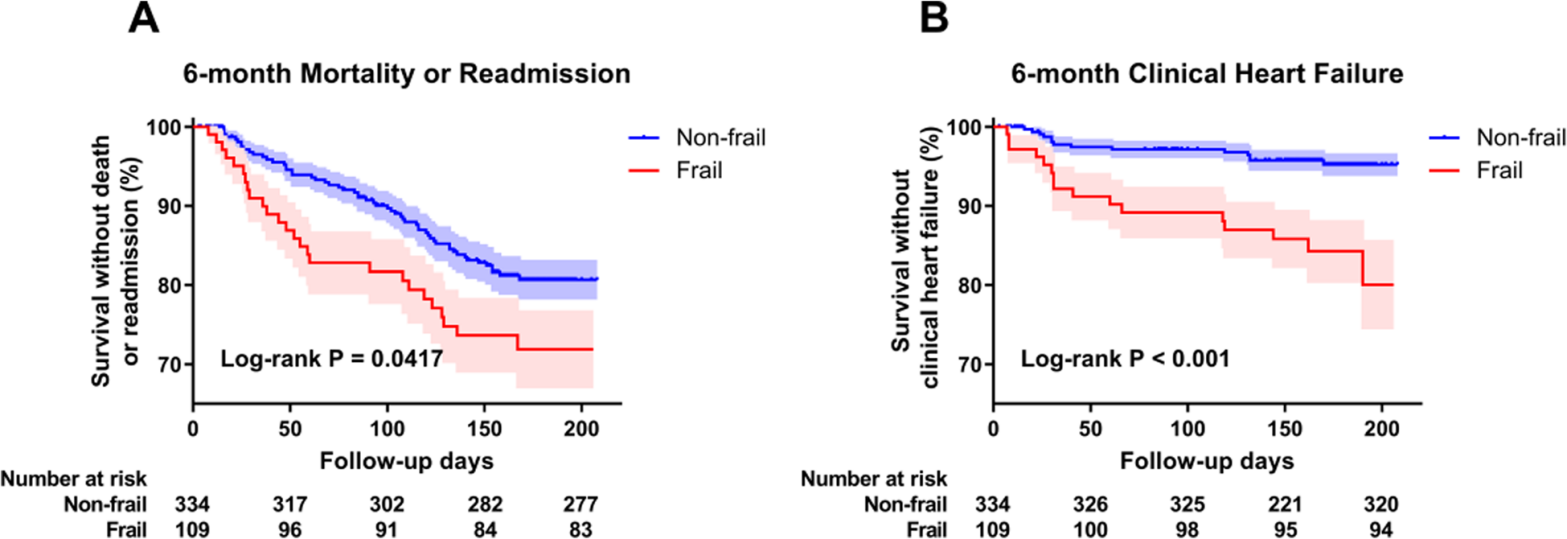
Kaplan-Meier survival curves by frailty in geriatric stage B heart failure inpatients. Frailty was defined by Fried frailty phenotype. Event rates of 6-month mortality or readmission (**a**) and 6-month clinical heart failure development (**b**) were analyzed by log-rank test

**Fig. 3:**
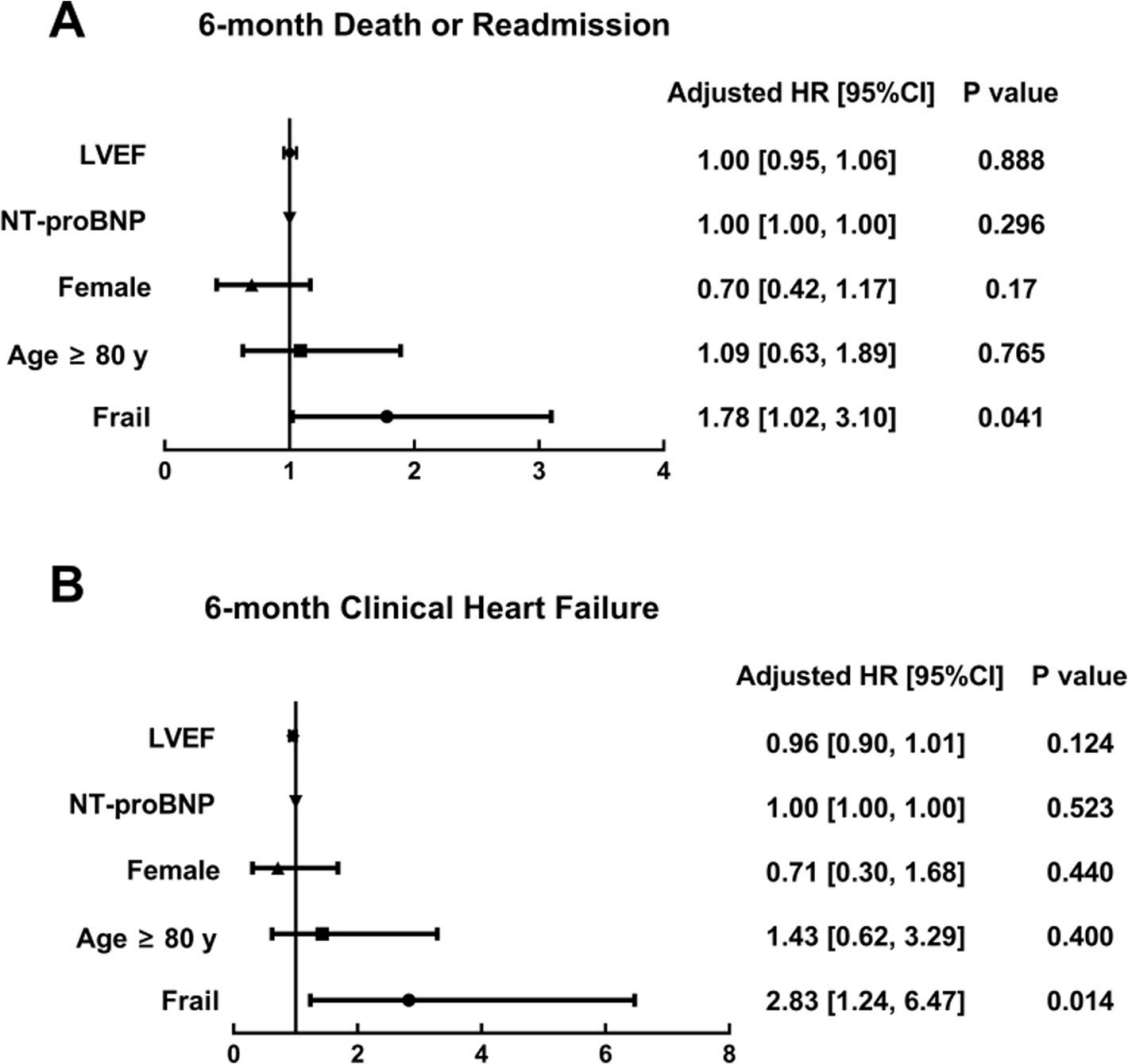
Multivariable Cox proportional hazard models of frailty, adjusted by age, sex, NT-pro BNP level, and LVEF. Frailty was defined by Fried frailty phenotype, and events were defined as 6-month mortality or readmission (**a**) and 6-month clinical heart failure development (**b**). HR, Hazard ratio; CI, confidence interval; LVEF, left ventricular ejection fraction

**Fig. 4:**
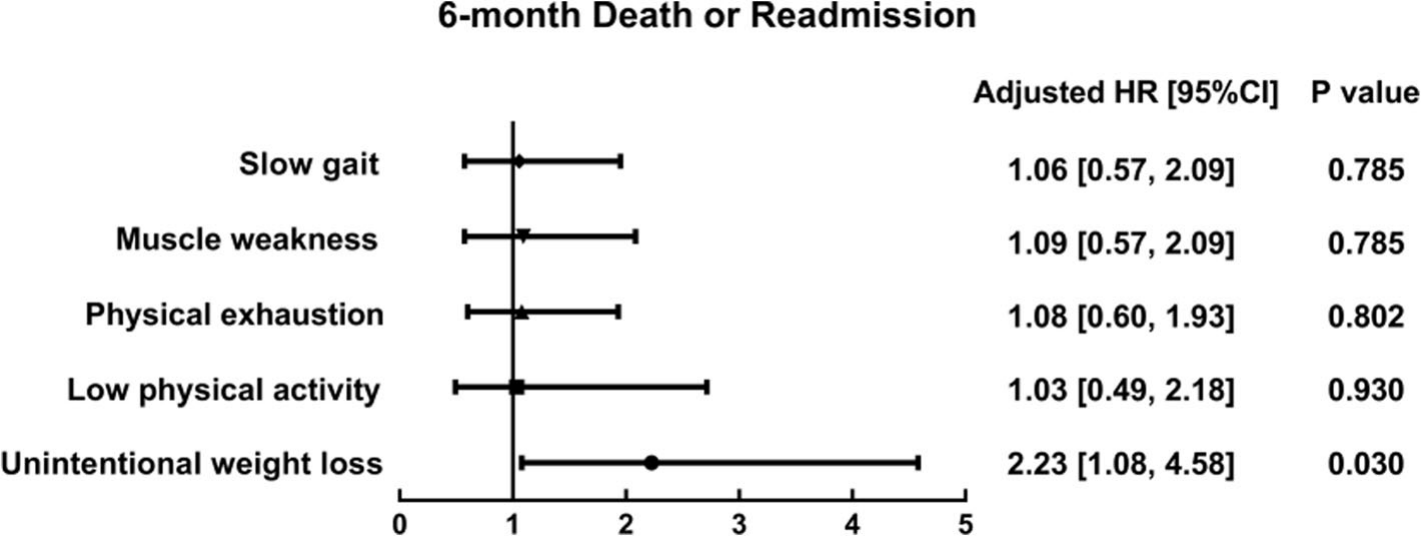
Multivariable Cox proportional hazard models of five frailty components, adjusted by age, sex, NT-pro BNP level, and LVEF. Frailty was defined by Fried frailty phenotype, and event was defined as 6-month mortality or readmission. HR, Hazard ratio; CI, confidence interval

During the 6-month follow-up, 26 (23.9%) in the frail group and 57 (17.1%) in the non-frail group experienced all-cause mortality or readmission. The mean survival time without mortality or readmission was 168 ± 6.8 days in the frail and 185 ± 2.9 days in the non-frail subjects (Fig. 2a*:* Log-rank *χ*^*2*^ = 4.15, *P* = 0.042). Frail individuals had a 1.78-times higher risk of mortality or readmission than non-frail individuals, independent of age, sex, NT-pro BNP level and LVEF (Fig. 3a*:* 95%CIs 1.02–3.10, *P* = 0.041). Among five frailty components, unintentional weight loss remained associated with a higher adjusted risk of 6-month mortality or readmission (Fig. 4).

### Clinical HF

During the 6-month follow-up, 15 (13.8%) participants in the frail group and 14 (4.2%) in the non-frail group developed clinical HF. The mean time for clinical HF development was 183 ± 5.6 days in the frail and 201 ± 1.8 days in the non-frail subjects (Fig. 2b*:* Log-rank *χ*^*2*^ = 13.61, *P* < 0.001). Frail individuals had a 2.83-times higher risk of developing clinical HF, independent of age, sex, NT-pro BNP level and LVEF (Fig. 3b*:* 95%CIs 1.24–6.47, *P* = 0.014).

## Discussion

This study explored the prognostic value of frailty, defined by the Fried frailty phenotype, in older SBHF inpatients. We found that frailty could independently predict the 6-month all-cause mortality or readmission and the development clinical HF in older inpatients.

Frailty was common among inpatients with SBHF - our study found that the prevalence of frailty diagnosed by the Fried phenotype was 24.6% in older SBHF inpatients, which was higher than that in people without HF (3%, aged 65–70 years) [17], and lower than that in clinical HF patients (32–76%) [6, 18]. A lower physical activity score, more comorbidities, and other factors in SBHF compared with healthy individuals [19] may account for the increased prevalence of frailty in older SBHF inpatients. Individuals with SBHF have fewer HF-related multisystem complications (systemic inflammation, comorbidity burden, older age, sarcopenic obesity, metabolic impairment, hemodynamic abnormalities, translocation of gut microbiome, etc.), and this along with other related factors [17] may account for the decreased prevalence of frailty in older SBHF inpatients.

Our study demonstrated that frail older SBHF inpatients were more likely to be older, had more comorbidities, lower LVEF, polypharmacy, depression, disability, and cognitive impairment. Among those, polypharmacy, disability, and cognitive impairment were independently associated with frailty. To a certain degree, our results matched the findings of previous studies in clinical HF patients, which also indicated an association between these factors and the development of frailty [5, 20–23]. The key mechanism underlying this association in clinical HF patients was related to worsening of the decreased reserve and resistance to stressors due to the inflammatory and metabolic disturbances caused by these factors [17]; this may also explain the similar phenomenon found in older SBHF inpatients. These factors should be screened to help identify older SBHF inpatients with a high risk of developing frailty.

We found that frailty could independently predict the 6-month all-cause mortality or hospital readmission in older SBHF inpatients, and this was unaffected by age, sex, NT-pro BNP level and LVEF. This finding was in agreement with the higher risk of readmission and mortality among frail patients with clinical HF [6, 24]. The prognostic value of frailty has been shown in patients with HF, and assessing frailty may help to identify patients with clinical HF who are at a higher risk of disability and adverse clinical outcomes at each stage of the disease [17].

We also explored the prevalence and predictive value of each frailty component in SBHF inpatients. Unintentional weight loss was the only component that predicted mortality or readmission which was different compared to the low physical activity component in clinical HF [6]. Previous studies have recognized the importance of unintentional weight loss in predicting poor outcomes [25]. Malignancy, cardiovascular disease, gastrointestinal disease, endocrinopathies, infectious disease, and psychiatric disease were the common causes of unintentional weight loss [25, 26]. All of the above mentioned causes may be related to higher mortality or readmission in SBHF inpatients. Furthermore, unintentional weight loss in older adults was connected to the physiology of aging and chronic medical conditions and also due to part decreases in lean muscle mass and increases in total body fat. In addition to this, sarcopenia was also prevalent. Sarcopenia is one of the important mechanisms of frailty [17]. Additionally, unintentional weight loss has no predictive value in clinical HF and this is probably due to inaccuracies resulting from its measurement, since edema and the use of diuretics directly influence body weight and are common in clinical HF.

Cardiac cachexia (the cachexia complicating HF) is often associated with anorexia, inflammation, and insulin resistance and is caused by a pathological shift in the balance between anabolism and catabolism and describes a state of unintentional weight loss that cannot be reversed by changes in nutritional intake alone [27]. Skeletal muscle is the most important functional tissue lost in the context of cachexia owing to catabolism and adipose tissue loss. In addition to sarcopenia, exercise capacity also decreased [28]. Cardiac cachexia, sarcopenia and unintentional weight loss are closely associated with frailty and worsen the outcomes of HF. Furthermore, the levels of serum adiponectin were significantly higher in patients with HF and cachexia, suggesting that adiponectin may play a critical role in cardiac remodeling in the presence of cachexia [29]. In particular, cachexia is also a risk factor for cardiovascular disease development. A potential therapeutic strategy in overlapping conditions of frailty, sarcopenia and cachexia included improving appetite, providing additional calories, and exercise training aimed at improving exercise capacity [28]. The treatment of cachexia may be a potential way to improve frailty in HF patients.

Although only 6.5% of older SBHF inpatients developed clinical HF during the follow-up period, we found that frailty could predict clinical HF independently, which may be due to the following reasons. Firstly, pre-clinical diastolic dysfunction predicts a markedly higher risk of progression to HF and death, and its prevalence increases with age, hypertension, atrial fibrillation/flutter, coronary artery disease, history of myocardial infarction, diabetes, and systolic dysfunction [30]. Secondly, exercise is very helpful from a symptomatic perspective, but frail individuals have a lower functional capacity. They more commonly have disabilities, experience falls, exercise ≤5 times per week, and are unable to walk 100 m without help.

Interestingly, the older SBHF inpatients in our study mainly had preserved LVEF and presented with structural and diastolic abnormalities, rather than systolic abnormalities. Since the evidence for interventions in patients with preserved ejection fraction is lacking and inconclusive, our results may suggest that better blood pressure control, exercise, and interventions targeting frailty may improve outcomes or reduce the risk of developing clinical HF in older SBHF inpatients. Nevertheless, it is difficult to achieve blood pressure control in these individuals, and this had been achieved in only half of our older SBHF inpatients with hypertension. Frailty is to a certain is a reversible condition. Interventions for hypertension, atrial fibrillation/flutter, cardiac rehabilitation, physical training, decreased polypharmacy (particularly involving unnecessary medication), and patient education might delay the progress of HF, or even reduce mortality [30].

There were several limitations to our study. First, our subjects were from a single tertiary hospital and none of the subjects had reduced LVEF, which might affect the generalizability of our results. Second, a gold standard measurement for frailty is lacking in China. Third, diabetes was associated with the outcome and the use of SGLT-2 was of great value. However, none of our participants were using SGLT-2, as SGLT-2 was not covered by medical insurance when we recruited the participants. Fourth, our follow-up period was not long enough and the rate of clinical HF development was low. Multicenter studies with a larger sample size, more information about cardiovascular risk factors, a longer follow-up period, and more meaningful and functional outcomes, like all cause death, all cause readmission, HF hospitalization, HF death, medical cost, institutionalization, prospective falls, prospective activity of daily living decline, and home time, are warranted in the future.

## Conclusions

Frailty is common among older SBHF inpatients. It can also predict the 6-month mortality or hospital readmission, and the development of clinical HF. Frailty status should be evaluated in older SBHF inpatients to help identify individuals with an increased risk of mortality or readmission, and developing clinical HF and interventions targeting frailty should be applied to help improve outcomes.

## Supplementary Information

**Additional file 1.** Criteria of Fried frailty phenotype.

**Additional file 2.** Factors associated with frailty by univariable and multivariable logistic regression analysis.

## Data Availability

All data analyzed during this study are included in this published article and its supplementary information files.

## Abbreviations

HF: Heart failure
SBHF: Stage-B heart failure
ARIC: Atherosclerosis Risk in Communities
GDS-5: Geriatric Depression Scale
HADS-A: Hospital Anxiety and Depression Scale
MMSE: Mini Mental State Examination
REDCap: Research Electronic Data Capture
ORs: Odds ratios
95%CIs: 95% confidence intervals
NT-pro BNP: N-terminal pro-B-type natriuretic peptide
LVEF: Left ventricular ejection fraction
HRs: Hazard ratios

## References

[1] Shah RU, Tsai V, Klein L, Heidenreich PA (2011). Characteristics and outcomes of very elderly patients after first hospitalization for heart failure. Circ Heart Fail..

[2] van Riet EE, Hoes AW, Limburg A, Landman MA, van der Hoeven H, Rutten FH (2014). Prevalence of unrecognized heart failure in older persons with shortness of breath on exertion. Eur J Heart Fail.

[3] Kojima G, Iliffe S, Walters K (2018). Frailty index as a predictor of mortality: a systematic review and meta-analysis. Age Ageing.

[4] Cameron ID, Fairhall N, Langron C, Lockwood K, Monaghan N, Aggar C (2013). A multifactorial interdisciplinary intervention reduces frailty in older people: randomized trial. BMC Med.

[5] Fried LP, Tangen CM, Walston J, Newman AB, Hirsch C, Gottdiener J (2001). Frailty in older adults: evidence for a phenotype. J Gerontol A Biol Sci Med Sci.

[6] Vidan MT, Blaya-Novakova V, Sanchez E, Ortiz J, Serra-Rexach JA, Bueno H (2016). Prevalence and prognostic impact of frailty and its components in non-dependent elderly patients with heart failure. Eur J Heart Fail.

[7] Goldfarb M, Lauck S, Webb JG, Asgar AW, Perrault LP, Piazza N (2018). Malnutrition and mortality in frail and non-frail older adults undergoing aortic valve replacement. Circulation..

[8] Yao SM, Zheng PP, Liang YD, Wan YH, Sun N, Luo Y (2020). Predicting non-elective hospital readmission or death using a composite assessment of cognitive and physical frailty in elderly inpatients with cardiovascular disease. BMC Geriatr.

[9] Yancy CW, Jessup M, Bozkurt B, Butler J, Casey DE, Drazner MH (2013). 2013 ACCF/AHA guideline for the management of heart failure: a report of the American College of Cardiology Foundation/American Heart Association task force on practice guidelines. J Am Coll Cardiol.

[10] van der Meer P, Gaggin HK, Dec GW (2019). ACC/AHA versus ESC guidelines on heart failure: JACC guideline comparison. J Am Coll Cardiol.

[11] Liang YD, Zhang YN, Li YM, Chen YH, Xu JY, Liu M (2019). Identification of frailty and its risk factors in elderly hospitalized patients from different wards: a cross-sectional study in China. Clin Interv Aging.

[12] Harris PA, Taylor R, Thielke R, Payne J, Gonzalez N, Conde JG (2009). Research electronic data capture (REDCap)--a metadata-driven methodology and workflow process for providing translational research informatics support. J Biomed Inform.

[13] Hoyl MT, Alessi CA, Harker JO, Josephson KR, Pietruszka FM, Koelfgen M (1999). Development and testing of a five-item version of the geriatric depression scale. J Am Geriatr Soc.

[14] Helvik AS, Engedal K, Skancke RH, Selbaek G (2011). A psychometric evaluation of the hospital anxiety and depression scale for the medically hospitalized elderly. Nord J Psychiatry.

[15] Wade DT, Collin C (1988). The Barthel ADL index: a standard measure of physical disability?. Int Disabil Stud.

[16] Folstein MF, Folstein SE, McHugh PR (1975). “Mini-mental state”. A practical method for grading the cognitive state of patients for the clinician. J Psychiatr Res.

[17] Pandey A, Kitzman D, Reeves G (2019). Frailty is intertwined with heart failure: mechanisms, prevalence, prognosis, assessment, and management. JACC Heart Fail..

[18] Jha SR, Hannu MK, Newton PJ, Wilhelm K, Hayward CS, Jabbour A (2017). Reversibility of frailty after bridge-to-transplant ventricular assist device implantation or heart transplantation. Transplant Direct.

[19] Gidding SS, Lloyd-Jones D, Lima J, Ambale-Venkatesh B, Shah SJ, Shah R (2019). Prevalence of American Heart Association heart failure stages in black and white young and middle-aged adults: the CARDIA study. Circ Heart Fail.

[20] Sze S, Zhang J, Pellicori P, Morgan D, Hoye A, Clark AL (2017). Prognostic value of simple frailty and malnutrition screening tools in patients with acute heart failure due to left ventricular systolic dysfunction. Clin Res Cardiol.

[21] Diaz-Toro F, Nazzal Nazal C, Verdejo H, Rossel V, Castro P, Larrea R (2017). Frailty in patients admitted to hospital with acute decompensated heart failure. Rev Med Chil.

[22] Sze S, Pellicori P, Zhang J, Weston J, Clark AL (2019). Identification of frailty in chronic heart failure. JACC Heart Fail..

[23] Pandey A, Kitzman D, Whellan DJ, Duncan PW, Mentz RJ, Pastva AM (2019). Frailty among older decompensated heart failure patients: prevalence, association with patient-centered outcomes, and efficient detection methods. JACC Heart Fail.

[24] Bottle A, Kim D, Hayhoe B, Majeed A, Aylin P, Clegg A (2019). Frailty and co-morbidity predict first hospitalisation after heart failure diagnosis in primary care: population-based observational study in England. Age Ageing.

[25] Perera LAM, Chopra A, Shaw AL (2021). Approach to patients with unintentional weight loss. Med Clin North Am.

[26] Bouras EP, Lange SM, Scolapio JS (2001). Rational approach to patients with unintentional weight loss. Mayo Clin Proc.

[27] Seferovic PM (2019). Introduction to the special issue entitled ‘Heart failure management of the elderly patient: focus on frailty, sarcopenia, cachexia, and dementia’. Eur Heart J Suppl.

[28] Bielecka-Dabrowa A, Ebner N, Dos Santos MR, Ishida J, Hasenfuss G, von Haehling S. Cachexia, muscle wasting, and frailty in cardiovascular disease. Eur J Heart Fail. 2020;22(12):2314–26. 10.1002/ejhf.2011. Epub 2020 Oct 14. PMID: 32949422. 10.1002/ejhf.2011 32949422

[29] Loncar G, Fulster S, von Haehling S, Popovic V (2013). Metabolism and the heart: an overview of muscle, fat, and bone metabolism in heart failure. Int J Cardiol.

[30] Wan SH, Vogel MW, Chen HH (2014). Pre-clinical diastolic dysfunction. J Am Coll Cardiol.

