# Supplementary material for: Frailty related all-cause mortality or hospital readmission among adults aged 65 and older with stage-B heart failure inpatients
Source: BMC Geriatr. 2021 Feb 16;21:125. doi: 10.1186/s12877-021-02072-6 (PMC7885474; doi:10.1186/s12877-021-02072-6)
Supplement: Supplementary file 1 — Additional file 1. Criteria of Fried frailty phenotype. [file 12877_2021_2072_MOESM1_ESM.docx]

| **Additional file 1. Criteria of Fried frailty phenotype.** | | |
| --- | --- | --- |
| **Item** | **Men** | **Women** |
| **Unintentional weight Loss** | Weight loss more than 5% regular weight or 4.5 kilograms unintentionally in the past year | |
| **Low Physical Activity**  Any of the described activities evaluated by short version of the Minnesota Leisure Time Activity questionnaire | ＜383kcal per week  Similar to taking a walk for less than 2.5 hours per week | ＜270kcal per week  Similar to taking a walk for less than 2 hours per week |
| **Physical exhaustion**  Any phenomenon described by the right questions occurred for 3 days or more | Assessed by self-report using the following questions:   1. How often in the last week did you feel that everything you did was an effort? 2. How often in the last week did you feel that you could not get going? | |
| **Muscle Weakness**  Grip strength measured by CAMRY electronic hand dynamometer for two times’ average | Average grip strength:  ≤29kg, BMI ≤24.0  ≤30kg, BMI 24.1-26.0  ≤30kg, BMI 26.1-28.0  ≤32kg, BMI ＞28.0 | Average grip strength:  ≤17kg, BMI ≤23.0  ≤17.3kg, BMI 23.1-26.0  ≤18kg, BMI 26.1-29.0  ≤21kg, BMI ＞29.0： |
| **Slowed Gait**  The faster speed to walk 4 meters for two times, with or without a walking aid | Walk speed:  ≤0.65m/s, Height ≤173cm  ≤0.76m/s, Height＞173cm： | Walk speed:  ≤0.65m/s, Height≤159cm：  ≤0.76m/s, Height＞159cm： |
| **Abbreviations:** BMI, body mass index. | | |
