# Supplementary material for: Frailty related all-cause mortality or hospital readmission among adults aged 65 and older with stage-B heart failure inpatients
Source: BMC Geriatr. 2021 Feb 16;21:125. doi: 10.1186/s12877-021-02072-6 (PMC7885474; doi:10.1186/s12877-021-02072-6)
Supplement: Supplementary file 2 — Additional file 2. Factors associated with frailty by univariable and multivariable logistic regression analysis. [file 12877_2021_2072_MOESM2_ESM.docx]

| **Additional file 2. Factors associated with frailty by univariable and multivariable logistic regression analysis.** | | | | | | | | |
| --- | --- | --- | --- | --- | --- | --- | --- | --- |
| Variables | Univariable analysis | | | | Multivariable analysis | | | |
|  | ORs | 95% CIs | | P values | ORs | 95% CIs | | P values |
|  |  | Lower | Upper |  |  | Lower | Upper |  |
| Age, years | 1.10 | 1.06 | 1.13 | **<0.001** | 1.03 | 0.98 | 1.09 | 0.209 |
| Living with spouse | 0.67 | 0.42 | 1.07 | 0.090 |  |  |  | - |
| Diastolic abnormality | 1.60 | 1.03 | 2.47 | **0.035** | 0.95 | 0.55 | 1.73 | 0.870 |
| LVEF | 0.96 | 0.92 | 1.00 | **0.048** | 0.99 | 0.94 | 1.04 | 0.587 |
| Log (NT-proBNP) | 2.60 | 1.55 | 4.37 | **<0.001** | 1.84 | 0.95 | 3.56 | 0.072 |
| Blood pressure controlled | 0.84 | 0.52 | 1.36 | 0.485 |  |  |  |  |
| ≥ 5 medications | 1.99 | 1.28 | 3.09 | **0.002** | 2.24 | 1.21 | 4.15 | **0.010** |
| GDS-5 ≥ 2 | 2.67 | 1.46 | 4.87 | **0.001** | 1.89 | 0.88 | 4.06 | 0.101 |
| HADS-A ≥ 8 | 2.33 | 0.91 | 5.94 | 0.078 |  |  |  | - |
| Barthel index < 60 | 4.47 | 1.83 | 10.92 | **0.001** | 4.88 | 1.05 | 22.63 | **0.043** |
| Fall in past year | 2.04 | 1.26 | 3.31 | **0.004** | 1.23 | 0.64 | 2.37 | 0.544 |
| MMSE < 24 | 4.213 | 2.48 | 7.16 | **<0.001** | 4.14 | 2.09 | 8.22 | **0.035** |
| Abbreviations: CIs, Confidence Interval; OR, Odds Ratio. Other abbreviations are the same as in Table2.  All covariates with a P value of less than 0.05 on univariable analysis were entered into the multivariable model. | | | | | | | | |
